# Diversity of helminths with zoonotic potential and molecular characterization of *Toxocara canis* infecting domestic dogs from locations of Amazon and Atlantic Forest Brazilian biomes

**DOI:** 10.1590/S1984-29612023078

**Published:** 2023-12-04

**Authors:** Tuan Pedro Dias-Correia, Leandro Batista das Neves, Fernanda Bittencourt-Oliveira, Gabriella Cristina Balzana Giglio, Thiago Cordeiro Pereira, Fernanda Barbosa de Almeida, Rosângela Rodrigues-Silva

**Affiliations:** 1 Laboratório de Referência Nacional em Hidatidose – LRNH, Laboratório de Parasitologia Integrativa e Paleoparasitologia – LPIP, Instituto Oswaldo Cruz – IOC, Fundação Oswaldo Cruz – Fiocruz, Rio de Janeiro, RJ, Brasil

**Keywords:** Zoonotic helminths, Toxocara canis, molecular characterization, mitochondrial genes, domestic dogs, Brazil, Helmintos zoonóticos, Toxocara canis, caracterização molecular, genes mitocondriais, cães domésticos, Brasil

## Abstract

The coproparasitological examination of dogs (n=278) from two Brazilian biomes (Amazon [AZ] and Atlantic Forest [AF]) by centrifugal flotation demonstrated positivity values of 54.2% (AF) and 48.5% (AZ). The most prevalent parasites in AF were hookworms (81.0% - 47/58), *Toxocara* sp. (17.3% - 10/58) and *Trichuris vulpis* (12.1% - 7/58); while in AZ they were hookworms (86.7% - 72/83), *Toxocara* sp. (18.1% - 15/83), *Dipylidium caninum* (13.3% - 11/83) and *T. vulpis* (10.8% - 9/83). PCR was performed using the partial mitochondrial genes cytochrome c oxidase subunit 1 (p*cox*1) and NADH dehydrogenase 1 (p*nad*1) in 25 fecal samples positive for *Toxocara* sp. eggs and found one sample positive for p*cox*1 and six positives for p*nad*1. The sequencing of these samples was unsuccessful due to the difficulties inherent in copro-PCR+sequencing. The sequencing of 14 samples of *T. canis* adult helminths retrieved 11 sequences of 414 bp for p*cox*1 and nine sequences of 358 bp for p*nad*1. The phylogenetic trees of these sequences confirmed the species *T. canis*. Intraspecific genetic variation was only observed for p*nad*1. This is the second study involving molecular analysis of *T. canis* in dogs from Brazil and adds new information through the use of p*nad*1.

## Resumo

O exame coproparasitológico de cães (n=278) de dois biomas brasileiros (Amazônia [AZ] e Mata Atlântica [MA]) por centrífugo-flutuação demonstrou valores de positividade de 54,2% (MA) e 48,5% (AZ). Os parasitos mais prevalentes na MA foram ancilostomídeos (81,0% - 47/58), *Toxocara* sp. (17,3% - 10/58) e *Trichuris vulpis* (12,1% - 7/58); enquanto na AZ foram ancilostomídeos (86,7% - 72/83), *Toxocara* sp. (18,1% - 15/83), *Dipylidium caninum* (13,3% - 11/83) e *T. vulpis* (10,8% - 9/83). A PCR foi realizada, utilizando-se os genes mitocondriais parciais da subunidade 1 do citocromo c oxidase (p*cox1*); e NADH desidrogenase 1 (p*nad1*) em 25 amostras fecais positivas para ovos de *Toxocara* sp. com uma amostra positiva para p*cox1* e seis positivas para p*nad1*. O sequenciamento dessas amostras não teve sucesso, devido às dificuldades inerentes ao copro-PCR+sequenciamento. O sequenciamento de 14 amostras de helmintos adultos *T. canis* recuperou 11 sequências de 414 pb para p*cox1* e nove sequências de 358 pb para p*nad1*. As árvores filogenéticas dessas sequências confirmaram a espécie *T. canis*. A variação genética intraespecífica foi observada apenas para p*nad1*. Este é o segundo estudo envolvendo análise molecular de *T. canis*, em cães do Brasil, e agrega novas informações com o uso do p*nad1*.

## Introduction

Since antiquity, dogs have been bred for companionship, hunting, protection, herding, and more recently in therapy programmes to provide support to human health (Dantas-Torres & Otranto, 2014; Sato et al., 2017). However, this close relationship with humans has favored the transmission of several pathogens with zoonotic potential (Dantas-Torres & Otranto, 2014).

The gastrointestinal helminths most frequently fund infecting dogs are hookworms, *Toxocara canis* (Werner, 1782) Stiles in Stiles & Hassall, 1905, *Trichuris vulpis* (Froelich, 1789) Smith, 1908, *Dipylidium caninum* (Linnaeus, 1758) and *Echinococcus* spp. Rudolphi, 1801 (Neves et al., 2017; Oliveira-Arbex et al., 2017; Ramos et al.; 2020; Silva et al., 2020; Lima et al., 2021; Souza et al., 2023; Ugalde et al., 2023). These parasites are associated with symptoms such as enteritis, diarrhea, vomiting, weight loss and anemia, among others, negatively affecting the development of dogs (Traversa, 2011).

The species *Toxocara canis* is the second most prevalent parasite in dogs, in addition to being the etiological agent of human toxocariasis. In endemic regions, the presence of *T. canis* and the disorders caused in dogs are indirect indications of the occurrence of this disease in humans (Sharma et al., 2015; Souza et al., 2023).

The coproparasitological findings in domestic dogs can be considered good indicators of environmental contamination, especially around the home, making it a low-cost tool for surveillance and control of zoonoses in the context of One Health (Delai et al., 2021; Souza et al., 2023).

The use of molecular techniques and automated sequencing for *Toxocara* species has been described mainly through the analysis of the internal transcribed spacer region (ITS) (Jacobs et al*.*, 1997) and mitochondrial genes (Li et al., 2008). However, there are few genetic characterization studies of *Toxocara* spp. and molecular data and phylogenetic analysis on isolates from Brazil are scarce (Mikaeili et al., 2015; Dantas-Torres, 2020).

Therefore, the objective of this work was to investigate the occurrence of helminths with zoonotic potential in domestic dogs from two different Brazilian regions and to carry out a molecular analysis of *Toxocara* sp. In these areas, this analysis can elucidate the role of domestic dogs in the transmission of helminthic zoonoses, since dogs can be sources of infection and sentinels of these infections (Salb et al., 2008).

## Materials and Methods

### Study areas

The survey was conducted in three different states of Brazil (Acre, Minas Gerais e Rio de Janeiro) in various expeditions carried out by the Laboratório de Referência Nacional em Hidatidose (LRNH-LPIP-IOC/Fiocruz) between 2014 and 2020. In the state of Acre, rural properties in settlements inside the Amazon Forest environment from five municipalities (Rio Branco, Bujari, Xapuri, Epitaciolândia, and Sena Madureira) were visited for sample collection. In the Conceição dos Ouros, Minas Gerais, the study location was farms near Atlantic Forest fragments. The study area in Rio de Janeiro (municipality of Rio de Janeiro) was the houses settled inside the Maciço da Pedra Branca/Parque Estadual da Pedra Branca, an Atlantic Forest biome conservation unit.

All the residences where the sample collection was done are inserted or near the forest environment of the Amazon (AZ) or Atlantic Forest (AF) biomes.

### Samples collection

A total of 278 fecal samples were collected from domestic dogs that interacted with the forest environment of the AZ (Acre, n=171) or AF (Minas Gerais [n=40] and Rio de Janeiro [n=67]) biomes. The dogs investigated were raised freely, having access to the both wild and peridomestic environment. Therefore, the owners did not have information about the dogs’ age. However, the vast majority were adults.

Adult *Toxocara* sp. worms were obtained from 12 abandoned dogs captured and housed at the Centro de Controle de Zoonoses Paulo Dacorso Filho (CCZ) and the Centro de Medicina Veterinária Jorge Vaitsman (CJV), both in the city of Rio de Janeiro, in 1993. After natural death, the dogs were necropsied for a previous study (Rodrigues-Silva et al. 1999) and the recovered helminths that were not of interest for that study (such as *Toxocara* sp.) were stored frozen (-20°C) in the LRNH.

### Coproparasitological examination

The fecal samples were fixed in 10% formalin and analyzed by the centrifuge-flotation method (Faust et al., 1938), mounted between slide and coverslip and observed by light microscopy to detect helminth eggs at 100x magnification. The eggs found were identified by their morphology. Replicates of the fecal samples fixed in 70% ethyl alcohol (when available) positive in the microscopy for *Toxocara* sp. were subjected to an egg concentration process by the improved flotation method using modified Breza solution (specific gravity = 1.4) (Széll et al., 2014). Subsequently, these eggs were recovered from flotation according to the protocol established by Öge et al. (2019), stored in 2 mL tubes and subjected to DNA extraction.

### Statistical analysis

The 95% confidence intervals (CI) for the proportions of the relative frequencies obtained from the coproparasitological examination were calculated using the software Excel 2019 (Microsoft Corporation, USA).

### DNA extraction

The *Toxocara* sp. eggs from fecal samples were subjected to DNA extraction using the QIAmp DNA Stool Mini Kit (Qiagen, Germany) with the modifications described by (Öge et al., 2019), and the DNA of the adult helminths was extracted using the QIAamp DNA Mini Kit (Qiagen, Germany) following the manufacturer’s instructions.

### PCR for pcox1 and pnad1 genes

The DNA samples were subjected to amplification of the partial region of the mitochondrial genes cytochrome c oxidase subunit I (p*cox*1) and NADH dehydrogenase 1 (p*nad*1) using the following primers: forward JB3 (5'-TTTTTTTGGGCATCCTGAGGTTTAT-3') and reverse JB4.5 (5'-TAAAGAAAGAACATAATGAAAATG-3'), used to amplify a p*cox*1 sequence of ≅ 450 bp (Bowles et al., 1992); and forward ND1F (5'-TTCTTATGAGATTGCTTTT-3') and reverse ND1R (5'-TATCATAACGAAAACGAGG-3'), for amplification of ≅ 370 bp p*nad*1 sequence (Li et al., 2008). All reactions were performed in a final volume of 25 µL containing 5-10 ng of template DNA, 10 mM Tris-HCL (pH 8.4), 50 mM KCL, 4 mM MgCl2, 200 µM of each dNTP, 50 pmol of each primer and 2 U Taq DNA polymerase (Invitrogen, USA). The thermal cycler parameters were initial denaturation at 94 °C for 5 min, 35 cycles of 94 °C for 30 s (denaturation), 50 °C for 30 s (annealing), and 72 °C for 30 s (extension), followed by a final extension step at 72 °C for 5 min (Li et al., 2008). PCR products were electrophoresed on 1% agarose gel in 1X TBE, stained with GelRed (Biotium, USA) and visualized with a UV transilluminator.

### Sequencing and phylogenetic analyses

PCR products were purified using Illustra GFX PCR DNA and the Gel Band Purification Kit (GE Healthcare, USA), following the manufacturer's instructions. Both DNA strands were sequenced using the same PCR primers and the Prim^TM^ ABI BigDye Terminator Cycle sequencing kit (Applied Biosystems, USA), according to the manufacturer’s protocol. Sanger sequencing of amplicons was performed with an automated DNA sequencer (ABI 3730 analyzer from Applied Biosystems, USA). Primer sequences were removed and a consensus sequence from the forward and reverse strands was assigned with SeqMan v. 7.1 (DNASTAR, Madison, USA) and then compared with the sequences available in the GenBank (NCBI, USA), with the Basic Local Alignment Search Tool (BLAST) (Altschul et al., 1990). 

**Figure 1:**
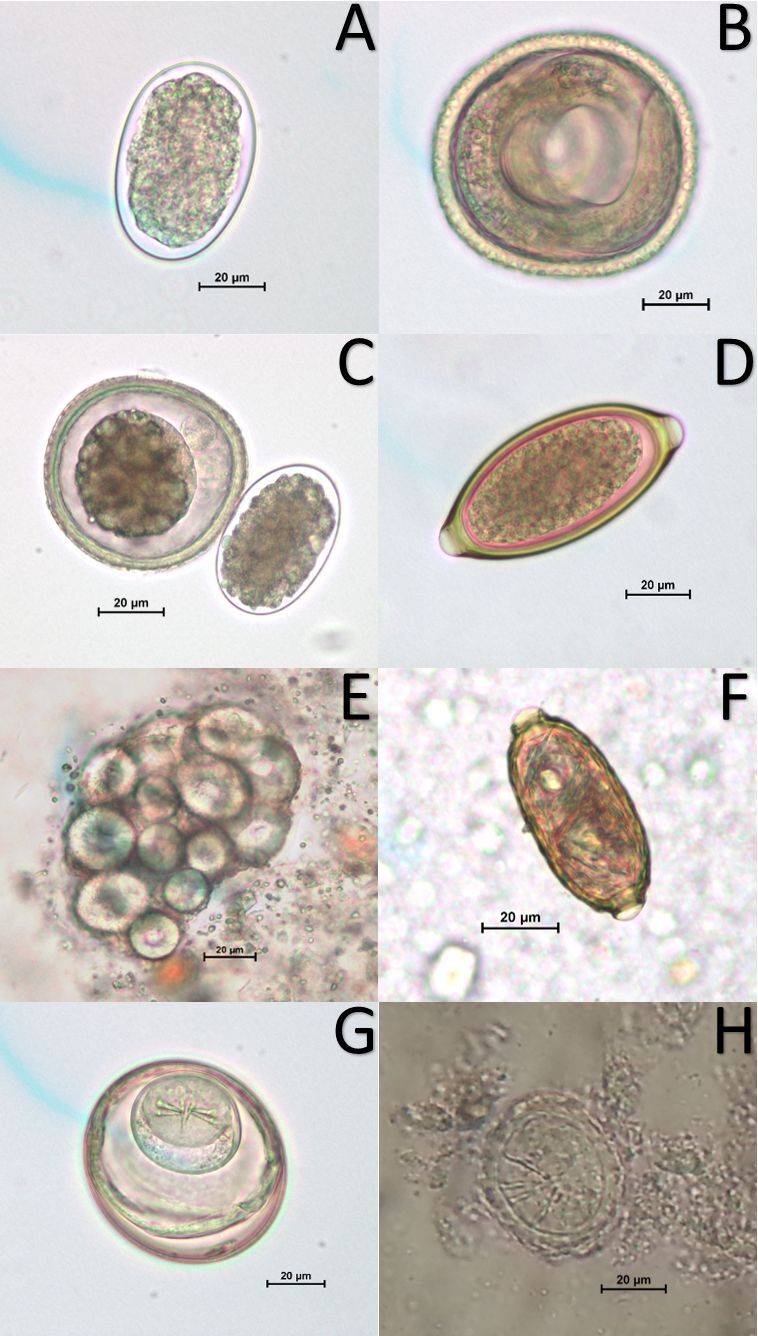
Photomicrographs of helminth eggs found in the parasitological examination domestic of dogs’ feces from the Atlantic Forest and Amazon biomes. (A) egg of hookworm; (B) egg of *Toxocara* sp.; (C) eggs of hookworm (right) and *Toxocara* sp. (left); (D) egg of *Trichuris vulpis*; (E) ovigerous capsule of *Dipylidium caninum*; (F) egg of capillarid (G) egg of *Rodentolepis nana*; and (H) egg of taenid.

**Figure 2:**
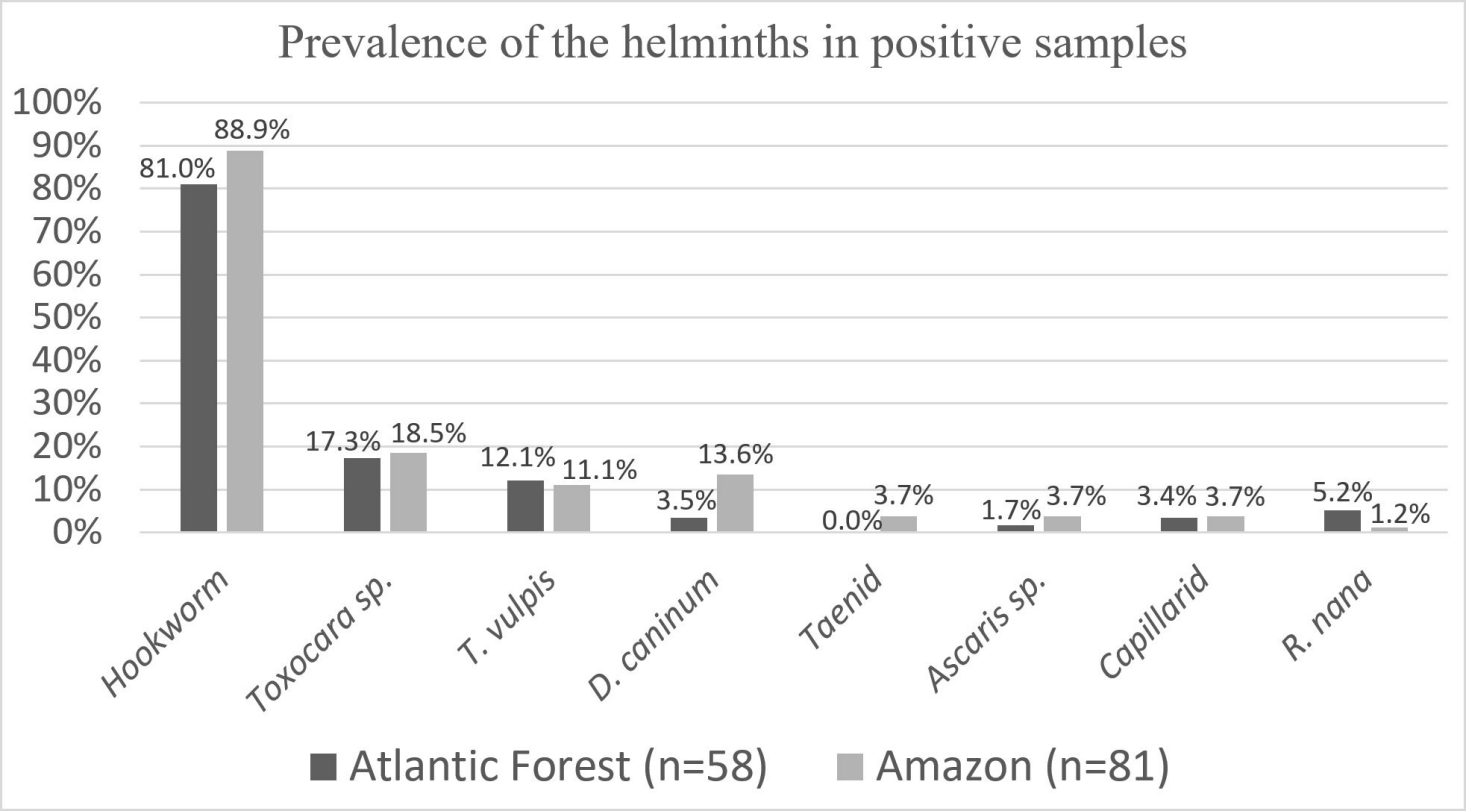
Prevalence of helminths in positive samples distributed according to the biome of origin.

Phylogenetic analyses were carried out with the MEGA v11.0 software (Tamura et al., 2021) based on alignment obtained from ClustalW of nineteen 414-bp sequences for p*cox*1 (11 generated in this study and 8 retrieved from the GenBank) and twenty 358-bp sequences for p*nad*1. The phylogenetic tree was constructed using the neighbor-joining method (Saitou & Nei, 1987) with the Kimura 2-parameter model of nucleotide substitution associated with gamma distribution (shape parameter = 1) (Kimura, 1980). Bootstrap analysis of 1000 replicates was applied (Felsenstein, 1985). All ambiguous positions were removed from each sequence pair (pairwise deletion option). The sequences used in the analysis were retrieved from the GenBank database and the accession numbers are shown in the trees (Figures 1 and 2). The *Ancylostoma caninum* (NC_012309) sequence was used as outgroup.

## Results and Discussion

### Coproparasitological examination of domestic dogs

Of the 278 fecal samples, 107 came from AF, with positivity for gastrointestinal helminths of 54.2% (58/107; CI: 44.8 – 63.7), and 171 came from AZ, with positivity of 48.5% (83/171; CI: 41.1 – 56.0). The occurrence for each helminth, both in single (monoparasitism) and mixed (polyparasitism) infections, is detailed in Table 1.

**Table 1:** Frequency of helminth eggs diagnosed in fecal samples of domestic dogs from the Amazon and Atlantic Forest biomes.

**Helminths**	**Amazon (n=171)**		**Atlantic Forest (n=107)**	
	**positive samples (%)**	**95% CI Lower -Upper**	**positive samples (%)**	**95% CI Lower-Upper**
Monoparasitism				
Hookworm	47 (27.49)	20.80 – 34.18	36 (33.64)	24.69 - 42.59
*Toxocara* sp.	6 (3.51)	0.75 – 6.27	4 (3.74)	0.14 - 7.34
*Trichuris vulpis*	2 (1.17)	0 – 2.78	3 (2.80)	0 - 5.93
*Dipylidium caninum*	0	-	1 (0.93)	0 - 2.75
Ascarid	0	-	1 (0.93)	0 - 2.75
Capillarid	1 (0.58)	0 – 1.72	0	-
*Rodentolepis nana*	1 (0.58)	0 – 1.72	2 (1.87)	0 - 4.44
Total of monoparasitism	57 (33.33)	26.26 – 40.40	47 (43.93)	34.53 - 53.33
Polyparasitism				
Hookworm + *Toxocara* sp.	6 (3.51)	0.75 – 6.27	5 (4.67)	0.67 - 8.67
Hookworm + *T. vulpis*	4 (2.34)	0.07 – 4.61	3 (2.80)	0 - 5.93
Hookworm + *D. caninum*	5 (2.92)	0.40 – 5.44	0	-
Hookworm + taenid	2 (1.17)	0 – 2.78	0	-
Hookworm + ascarid	1 (0.58)	0 – 1.72	0	-
Hookworm + capillarid	1 (0.58)	0 - 1.72	0	-
*Toxocara* sp. + *D. caninum*	1 (0.58)	0 - 1.72	0	-
Hookworm + *Toxocara* sp. + *T. vulpis*	0	-	1 (0.93)	0 - 2.75
Hookworm + *Toxocara* sp. + *D. caninum*	1 (0.58)	0 - 1.72	0	-
Hookworm + *T. vulpis* + taenid	1 (0.58)	0 - 1.72	0	-
Hookworm + *D. caninum* + capillarid	1 (0.58)	0 - 1.72	1 (0.93)	0 -2.75
Hookworm + *T. vulpis* + *D. caninum*	1 (0.58)	0 - 1.72	0	-
Hookworm + capillarid + *R. nana*	0	-	1 (0.93)	0 - 2.75
Hookworm + *T. vulpis* + *D. caninum* + ascarid	1 (0.58)	0 - 1.72	0	-
Hookworm + *Toxocara* sp. + *D. caninum* + ascarid	1 (0.58)	0 - 1.72	0	-
Total of polyparasitism	26 (15.20)	9.82 - 20.58	11 (10.28)	4.53 - 16.03

CI: confidence intervals.

Of the helminth eggs found (Figure 1), the most frequent in AF were hookworms (81.0%; 47/58; CI: 70.9 – 91.1), *Toxocara* sp. (17.3%; 10/58; CI: 7.5 – 27.0) and *Trichuris vulpis* (12.1%; 7/58; CI: 3.7 – 20.1) (Figure 2). Regarding AZ, the prevalence levels were hookworms (86.7%; 72/83; CI: 79.5 – 94.0), *Toxocara* sp. (18.1%; 15/83; CI: 9.8 – 26.4), *Dipylidium caninum* (13.3%; 11/83; CI: 6.0 – 20.5) and *T. vulpis* (10.8%; 9/83; CI: 4.2 – 17.5) (Figure 2).

In both regions, the most prevalent helminths were hookworms, followed by *Toxocara* sp. (Figure 2). This prevalence levels have been reported in several previous studies in Brazil (Oliveira-Arbex et al., 2017; Ramos et al.; 2020; Silva et al., 2020; Lima et al., 2021; Souza et al., 2023; Ugalde et al., 2023) and other countries, such as Mexico (Torres-Chablé et al., 2015), Algeria (Ziam et al., 2022), Nepal (Sukupayo & Tamang, 2023), Morocco (Idrissi et al., 2022) and Ecuador (Calvopina et al., 2023). Since most of the dogs investigated are adults, this data aligns with the literature, as infections by hookworms are the most common in adult dogs (over one year old) (Ferreira et al., 2016; Lima et al., 2021; Souza et al., 2023), and *Toxocara* sp. is a parasite more often found infecting puppies, although the infection can occur at any age (Villeneuve et al., 2015; Souza et al., 2023).

Although it has no known zoonotic potential, the nematode *T. vulpis* is among the most prevalent helminth parasites of domestic dogs with significant frequencies (Figure 2). The notable prevalence of this parasite in dogs is found in other similar studies (Silva et al., 2022; Souza et al., 2023; Ugalde et al., 2023).

The results also demonstrate the presence of *Rodentolepis nana* (Siebold, 1852), capillarids and *Ascaris* spp., but these parasites are not commonly found infecting domestic dogs (Woodland, 1924), which may explain the low prevalence found.

*R. nana* eggs were also found in studies involving the feces of wild carnivores (wolves and foxes), and the authors suggested that the finding may have been due to the ingestion of infected rodents or even eggs laid in the environment by definitive hosts (Elmore et al., 2013; van Kesteren et al., 2015).

Oliveira et al. (2022) demonstrated humans being act as carriers of capillarid eggs (spurious infection) in the same locations in the Amazon region analyzed here. Likewise, the capillarid eggs reported may have been ingested through the consumption of the viscera of parasitized predated/hunted rodents, as Neves et al. (2017) report dogs from the same areas in the state of Acre being fed with this type of material.

The low prevalence of taenid eggs in canine fecal samples has also been reported in other studies (Yamamoto et al., 2006; Sager et al., 2006; Antolová et al., 2009; Neves et al., 2017). Although the parasitological analysis does not allow distinguishing the species, numerous works of analysis of dog feces that found taenid eggs identified as *Echinococcus* sp. in other countries (Yu et al., 2008; Dyachenko et al., 2008; Bružinskaitė et al., 2009; Nagy et al., 2011) and in Brazil (Neves et al., 2017).

When observing the frequency of helminths in the biomes (Figure 2), it is possible to notice an apparent homogeneity since the difference between biomes for each parasite is small (around 10% maximum) and the CI values coincide. This may be associated with the historical connection bridges between these two biomes. Biogeography studies of small mammals demonstrate this flow of animals during evolutionary history, which probably resulted in the overlap of taxa in these biomes (Costa, 2003). Silva et al. (2022) also reported a relatively high prevalence (7.7%) of *D. caninum* infecting dogs living in the Atlantic Forest biome compared to other parasitological survey studies. It is known that helminth eggs can survive for long periods in moist and shaded soil (Silva et al., 2022). However, more studies are needed to understand the factors that lead to this prevalence in forest areas and the phylogeography of these taxa in Brazil.

Coproparasitological surveys in dogs are extremely important in the context of One Health. In Brazil, studies of this nature are concentrated in the Southeast region (Dantas-Torres, 2020), making it difficult to truly understand the distribution of these parasites in a country of continental proportions such as Brazil (Dantas-Torres & Otranto, 2014). Data are even scarcer in the case of rural dogs with access to forest areas, which can act as sources of infection in humans and other wild animals (Curi et al, 2017; Silva et al., 2022). In this regard, this study ratifies the circulation of these parasites in Brazil’s North region, more specifically in the state of Acre. Additionally, a recent study brought important information about the risk of emergence of zoonoses in Brazil, finding Acre to be the state with the highest risk factor according to the analyzed variables (distance from the city, richness of mammals, natural vegetation cover and deforestation, among other factors) (Winck et al., 2022), highlighting the attention needed for this state.

The prevalence of gastrointestinal parasites in dogs reported here, mainly hookworms and *Toxocara* sp., is in line with the findings of other studies that have demonstrated the need for a surveillance program in Brazil, along the lines of One Health concept, for the prevention of zoonotic diseases transmitted by dogs that circulate between domestic and wild areas, in order to avoid possible spillover events (Dantas-Torres & Otranto, 2014; Curi et al., 2017; Silva et al., 2022; Winck et al., 2022).

### PCR and *Toxocara* sp. sequencing

Twenty-five fecal samples were positive for the presence of eggs compatible with *Toxocara* sp. After submitting these samples to PCR, one amplified the target fragment of ≅ 450 bp of the p*cox*1 gene while six 6 amplified the target fragment of ≅ 370 bp of p*nad*1. However, attempts at sequencing the fecal samples were unsuccessful. In a study carried out in Turkey, the authors reported the same low effectiveness of PCR in identifying *Toxocara* sp. eggs compared to sedimentation/flotation techniques (5/21, or 23.8%) (Öge et al., 2019), values close to those of this study (7/25, or 28%). The effectiveness of PCR in identifying eggs in feces is directly related to the number of eggs available, making it difficult to use as a routine diagnostic tool for canine ascarid infections, given the low egg concentration (Öge et al*.*, 2019). Another possible factor is the resistance of ascarid eggshells due to their lipid composition (ascaroside), which covers the inner surface of the chitinous layer (Venkatesan et al., 2022).

Of the 14 adult helminth isolates that were positive by PCR, 11 sequences of 414-bp for the p*cox*1 gene were sequenced (OR004956-OR004966), while 9 sequences of 358-bp were obtained for the p*nad*1 gene (OR088860-OR088868). The sequences for p*cox*1 showed no variation in size or nucleotide composition and the A+T contents were 62%. Among the p*nad*1 sequences, the only one that showed nucleotide differences was the isolate H.A.13 (OR088868) at positions 35, 186, 286 and 322 (totaling 4 bp). The nucleotide composition of A+T for p*nad*1 ranged from 64.2 to 64.8%. Values close to A+T contents for mitochondrial (mtDNA) genes have been reported in other studies, and the results are compatible for nematodes (Li et al., 2008; Chen et al., 2022).

### Phylogenetic analysis

**Figure 3:**
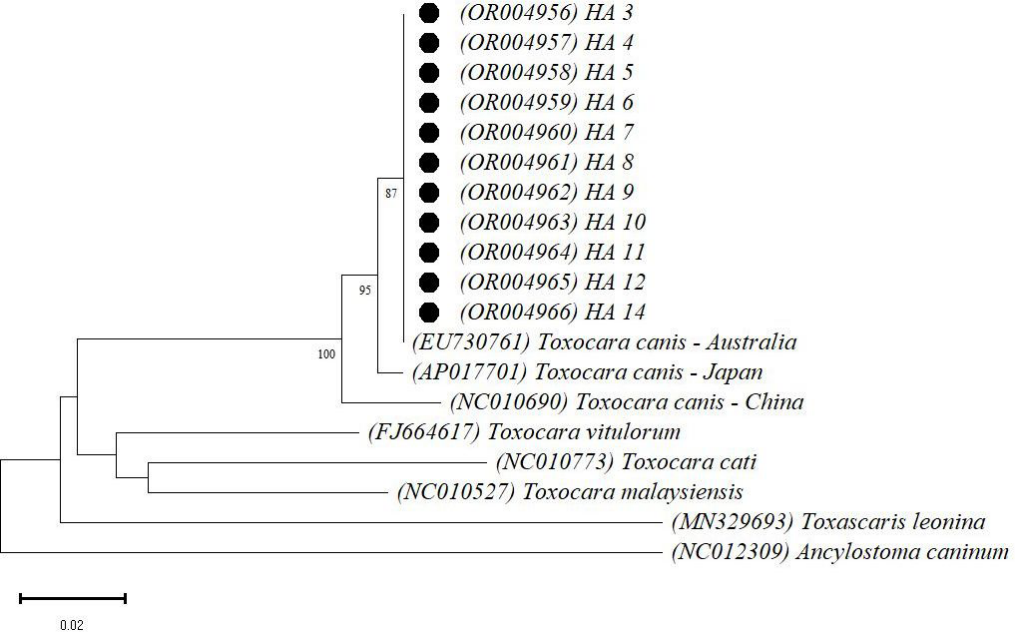
Phylogenetic relationship based on 414-pb of p*cox1* sequences of *Toxocara canis* isolates. Only bootstrap values above 60 are shown. The scale bar indicates the number of base substitutions per site: 0.02. The GenBank accession numbers from the sequences used are between parentheses and samples from this study are marked with a black circle.

**Figure 4:**
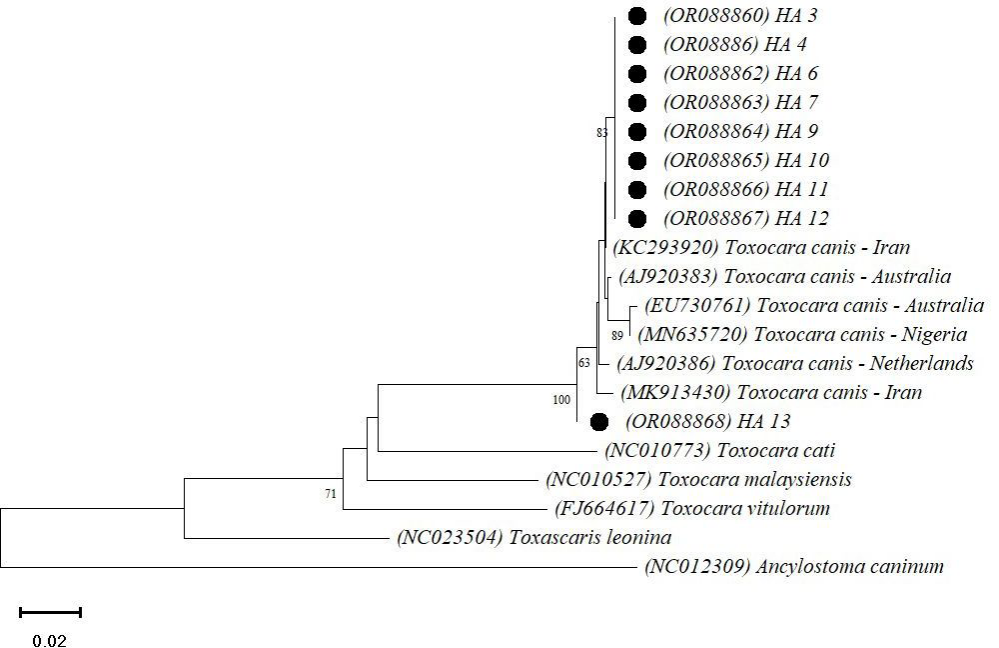
Phylogenetic relationship based on 360-pb of pnad1 sequences of *Toxocara canis* isolates. Only bootstrap values above 60 are shown. The scale bar indicates the number of base substitutions per site: 0.02. The GenBank accession numbers from the sequences used are between parentheses and samples from this study are marked with a black circle.

The phylogenetic trees (Figures 3, 4) demonstrated the formation of a well-supported clade between the newly generated sequences and the GenBank reference sequences for *T. canis* from different countries, confirming the species of the adult specimens from dogs of Rio de Janeiro, Brazil. The genetic distance (p-distance) between the *T. canis* samples in the study for p*cox*1 and the sequence from Australia (EU730761) was zero, in a sample that showed 100% homology by BLAST. Compared with the sequences used in the p*cox*1 tree (Figure 3), there was a variation of 0.01 for the sample from Japan (AP017701) and 0.03 for the samples from China (NC010690). The p-distance values related to other species of the genus *Toxocara* used in the construction of the p*cox*1 tree were approximately 0.14 (*Toxocara cati*), 0.12 (*Toxocara malaysiensis*), and 0.11 (*Toxocara vitulorum*), while for *Toxascaris leonina*, another ascarid prevalent in canids, it was 1.80.

For the p*nad*1 tree (Figure 4), the p-distance values between studied sequences ranged from zero to 0.013. With the exception of isolate H.A.13 (OR088868), p-distance values of the p*nad*1 sequences compared with other countries was 0.009 for Netherlands (AJ920386), 0.003-0.011 for Iran (KC293920 and MK913430), 0.006-0.014 for Australia (AJ920383 and EU730761) and 0.012 for Nigeria (MN635720). The H.A.13 (OR088868) had p-distance values between 0.011 and 0.020 with all other *T. canis* samples used in the phylogenetic reconstruction. In general, the variations between the species of the genus were 0.137-0.211 for p*nad*1.

Intraspecific genetic variations for *T. canis* using mtDNA genes have been reported with values close to 1.3% in several studies (Li et al., 2008; Mikaeili et al., 2015; Fava et al., 2020). Similar to our results, Mikaeili et al. (2015) found slightly higher values with the use of the p*nad*1 gene compared to p*cox*1 (0-1.3% and 0-1.7%, respectively). In contrast, results by Li et al. (2008) demonstrated the opposite, with greater nucleotide differences for p*cox*1.

In the present study, the genetic characterization was performed focusing on mtDNA, more specifically on the p*cox*1 and p*nad*1 genes. Mitochondrial markers have proven to be a useful tool for the investigation of the phylogeny of different helminths, including toxocarids (Li et al., 2008; Mikaeili et al., 2015; Öge et al., 2019; Fava et al., 2020). They have been used in recent studies to solve nematode systemic problems (Deng et al., 2022; Nisa et al., 2022). In addition to the mitochondrial genes used here, the use of markers for mitochondrially encoded ATP synthase membrane subunit 6 (ATP 6) (Wickramasinghe et al., 2009), NADH dehydrogenase subunit 4 (nad4) (Li et al., 2008) and ITS (Jacobs et al., 1997) genes have also been reportedly successful in the discrimination of species and phylogenetic analysis of the genus *Toxocara*.

To the best of our knowledge, there is only one study (Fava et al., 2020) that molecularly analyzed *T. canis* isolates from Brazilian dogs, and the researchers used only the partial p*cox*1 gene. Thus, our study is the second to carry out molecular characterization of *T. canis* isolates from Brazil, and adds information through the use of p*nad*1 in combination of p*cox1*, contributing molecular data in a scenario of scarcity.

In conclusion, different taxa of parasites with zoonotic potential with high prevalence were found in samples of feces from dogs from the states of Acre, Minas Gerais and Rio de Janeiro, alerting to the risk of human infection. It is evident that dogs play a fundamental role as a sentinel in the epidemiological dynamics of these zoonoses, thus requiring attention in prevention and control efforts, especially in rural/forest regions. To obtain a better understanding of *T. canis* in Brazil through knowledge of the parasite-host dynamics and the genetic diversity between populations, it is necessary to study more isolates from different geographic regions.
